# Verbal Episodic Memory and Endogenous Estradiol: An Association in Patients with Mild Cognitive Impairment and Alzheimer's Disease

**DOI:** 10.1155/2011/673012

**Published:** 2011-12-19

**Authors:** D. M. Bittner, V. Bittner, M. W. Riepe

**Affiliations:** ^1^Memory Clinic and Department of Neurology, University of Ulm, 89075 Ulm, Germany; ^2^Department of Neurology, Otto-von-Guericke University of Magdeburg, 39120 Magdeburg, Germany; ^3^Psychiatry II Department of Gerontopsychiatry, University of Ulm, 89321 Ulm, Germany

## Abstract

In the continuum of patients with Alzheimer's disease (AD), mild cognitive impairment (MCI), and normal controls, a possible association of verbal memory and endogenous estradiol (E_2_) levels was investigated. Verbal episodic memory was measured with a german version of the California verbal memory test (CVLT). Results were controlled for apolipoprotein E (ApoE) phenotype. We studied 37 controls, 32 MCIs and 117 ADs. Groups differed in all trials of the CVLT (*P* < 0.001) and in E_2_ levels (*P* < 0.001). E2 levels differed significantly between groups only among females (*P* < 0.001). In females correcting for age and ApoE, there was an overall correlation between CVLT delayed recall and level of E_2_ (*P* = 0.025). Stepwise regression analyses found E_2_ level to be a significant predictor for CVLT delayed recall (*P* < 0.001). It may be concluded that lower E_2_ levels occur more in the course of the disease than may be considered as a risk factor per se.

## 1. Introduction

Alzheimer's dementia is the most frequent dementia. Clinically, well-established criteria have proven their overall accuracy and usefulness [1]. Besides a characteristic neuropsychological course of the disease [2], several biological markers have been proposed including characteristic patterns of blood flow and glucose utilization [3–6], an increase of tau protein levels in cerebrospinal fluid, and a decrease of amyloid beta (1–42) protein [7, 8]. Also, in patients with Alzheimer disease, an increase of endogenous glucocorticoids was found [9]. Increased cortisol levels affect hippocampal neuron survival and potentiate beta-amyloid toxicity. Similarly, under experimental conditions, neuroprotection was found upon exogenous application of estradiol [10], most likely through a reductive effect of the amyloid-beta-induced toxicity [11].

Estradiol experimental studies have shown that estradiol may be neuroprotective in Alzheimer's disease (AD) and reduces amyloid toxicity [12]. Epidemiological evidence suggested that hormone replacement therapy might be beneficial in aged women [13, 14] but the results are still under dispute [15]. However, in a more recent large placebo-controlled, double-blind study including 4532 postmenopausal women not only no positive effect of a combination of estrogen and progestin [16] or estrogen alone on cognition but even a higher incidence of, AD was observed. Contrary in another but only small placebo-controlled, double-blind trial in manifest AD women estradiol demonstrated a significant effect on verbal and visual memory and attention compared with placebo [17]. Even less is known about endogenous estrogen levels in aging and dementia. In a recent study, lower E_2_ levels were correlated with poor cognition, behavioral and functional status in older individuals and AD patients [18]. A controversially result was reported by Cunningham et al. [19] in a cross-sectional study where neither a difference in estradiol levels between AD women and controls nor a correlation to cognitive tests was observed. However, estradiol may decline in women that develop AD [20].

With higher age, estradiol levels decrease in females and males [21, 22]. It was thus a goal of the current study to investigate whether endogenous estradiol in Alzheimer's disease is similar to aged controls and mild cognitive impairment as defined by Petersen et al. [23] and whether there is an association to verbal memory, the hallmark of AD's neuropsychological deficit.

## 2. Material and Methods

### 2.1. Subjects and Patients

Subjects were recruited from the Memory Clinic, Department of Neurology, University of Ulm. Of 190 consecutive subjects, 4 were excluded because of current estrogen replacement therapy. The remaining 186 were aged 35 to 89 years, 83 were men (age 35 to 88 years, median ± SD: 66.9 ± 10.27 y), and 103 were women (age 44 to 89 years, median ± SD: 70.2 ± 9.6 y). Medical histories were obtained and physical examinations performed. Alzheimer's disease was diagnosed using the NINCDS-ADRDA [1]. Mild cognitive impairment (MCI) was diagnosed according to the criteria by Petersen et al. [23]. 32 were classified as MCI (age 65.5 ± 6.9 y, 9 females and 23 males, MMST 28.1 ± 1.7) and 117 as AD (age 72.0 ± 8.3 y, 77 females and 40 males, MMST 21.9 ± 5.6), while there were 37 controls (age 60.7 ± 11.3 y, MD ± SD, 17 females and 20 males, MMST 29.2 ± 1.2). Controls were recruited from the memory clinic as well, admitted for subjective memory complaints or because of a positive family history where a cognitive impairment could be ruled out.

### 2.2. Neuropsychological Testing

The Mini-Mental State Examination [24] was used to assess cognitive functioning. In the verbal episodic memory task (California verbal memory test (CVLT)), five consecutive trials of the same list have to be remembered followed by a short (CVLT SD) and a long delayed recall (CVLT LD) as well as a recognition condition (CVLT rec) [25].

### 2.3. Clinical Chemistry

Blood samples were collected via venipuncture. Analysis of samples was conducted within 1 month of blood being drawn. Levels of E_2_ were measured by radioimmunoassay (double antibody technique) using a commercial kit.

### 2.4. Statistics

Statistical analysis was performed using a software program (SPSS 11.0; SPSS Inc, Chicago, Ill). Analysis of variance was used to test for heterogeneity with Tukey-B post hoc analysis for significant group differences followed by an ANCOVA where variables significantly different between groups were included. Spearman correlation coefficients were used where applicable to assess an association among measures. A stepwise regression was applied to assess the main contributing factors for verbal memory functioning.

## 3. Results

190 subjects were investigated, and 4 were excluded because of current estrogen replacement therapy. The remaining 37 control subjects, 32 MCIs and 117 patients with Alzheimer's disease were different in sex distribution, apolipoprotein E (ApoE) *ε*4 carriers, age, and general intelligence (Table 1), thus, in further analyses, data were controlled for age, ApoE *ε*4, and gender.

To assess for group differences of E_2_ levels, analyses of variance (ANOVA) were applied that revealed a significant, difference (F(2, 183) = 12.6, *P* < 0.001) indicating an impaired cognitive status to be associated with a decreased E_2_. Tukey post hoc analyses revealed only significant differences between the controls and AD (mean difference: 18.3 ± 3.8 pg/mL, *P* < 0.001, 95%-CI: 9.5–27.3 pg/mL), but not MCI (*P* = 0.117). Between AD and MCI difference was almost significant (mean difference: 8.8 ± 4.0 pg/mL, *P* = 0.07, 95%-CI: 18.2–0.6 pg/mL). Additionally, group differences in the CVLT were observed (Table 1).

**Figure 1:**
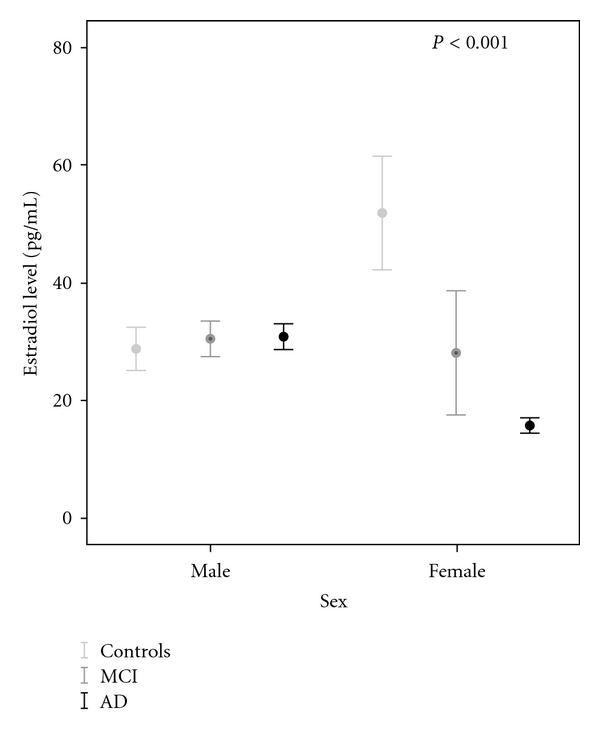
Differences of estradiol levels in controls, MCI, and AD patients (gender considered separately).

When the group comparison was stratified for gender, there were no differences of E_2_ for men (F(2, 80) =.2, *P* > 0.05) but for women (F(2, 99) = 20.9, *P* < 0.001). In Tukey post hoc, AD (mean difference: 36.2 ± 5.6 pg/mL, *P* < 0.001, 95%-CI: 49.6–22.8 pg/mL) and MCI (mean difference: 23.9 ± 8.7 pg/mL, *P* = 0.019, 95%-CI: 44.5–3.2 pg/mL) had lower E_2_ level than controls. There were no differences in the E_2_ level between MCI and AD (Figure 1).

To find out if lower E2 is independently associated to AD and MCI diagnosis in further analyses, an ANCOVA comparison of E_2_ between groups adjusted for significant noncognitive variables was performed. After including age, gender, and ApoE status, the model remained significant (F(7, 162) = 8.2, *P* < 0.001) with group (*P* < 0.001) and age (*P* < 0.001) as significant independent variables. Also, the interaction group *x* age was significant (*P* < 0.001).

**Figure 2:**
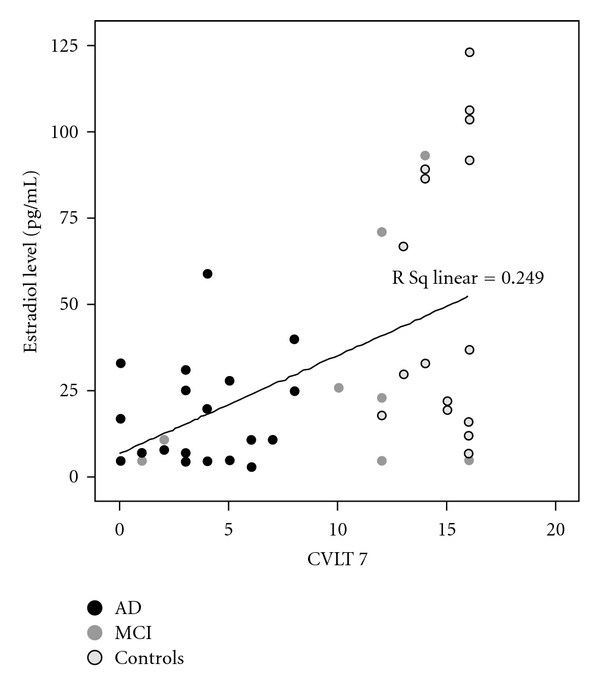
Association of estradiol level and CVLT delayed recall for all female subjects.

Spearman correlation showed a relation between estrogen and MMSE (*r* = 0.273,  *P* < 0.001), CVLT short delay (SD) (*r* = 0.197, *P* = 0.036), and CVLT delayed recall (LD) (*r* = 0.208, *P* = 0.026). There was no association of CVLT recognition and estrogen (*r* = .121, *P* = 0.2). When analyses was stratified for gender, there was no association of E_2_ with MMSE (*r* = .06, *P* > 0.05), CVLT SD (*r* = −.05, *P* > 0.05), CVLT LD (*r* = −.05, *P* > 0.05), and CVLT rec (*r* = .06, *P* > 0.05) in men. In women, a lower E_2_ was associated with a worse MMSE (*r* = .249, *P* = 0.017), CVLT SD (*r* = .417, *P* = 0.004), and CVLT LD (*r* = .428, *P* = 0.003; Figure 2). There was a tendency for a lower E2 to be associated with an impaired CVLT rec (*r* = .28, *P* = 0.06).

In univariate analysis with CVLT scores and MMSE as dependent variables, MMSE (F(1, 171) = 14.6, *P* < 0.001), CVLT SD (F(1, 171) = 9.4, *P* = 0.003), CVLT LD (F(1, 171) = 11.7, *P* = 0.001), and CVLT rec (F(1, 171) = 4.8, *P* = 0.03) were significantly associated with E_2_. In a linear stepwise regression analyses, correcting for age, gender and, ApoE E_2_ turned out to be a significant predictor of CVLT SD (F(1, 171) = 9.8, *r* = 0.408, *P* < 0.001) and CVLT LD (F(1, 171) = 9.7, *r* = 0.407, *P* < 0.001). Stratified for gender, there was no association in men for MMSE (*P* > 0.05), CVLT SD (*P* > 0.05), CVLT LD (*P* > 0.05), and CLT rec (*P* > 0.05). In women, regression analysis revealed CVLT LD only to be a significant predictor of E2 level (F(1, 79) = 17.9, *P* < 0.001), while there was no significant association for CVLT SD (*P* > 0.05), CVLT rec (*P* > 0.05), and MMSE (*P* > 0.05).

## 4. Discussion

We found an association between endogenous estrogen levels and memory functioning. This preliminary study showed that in, AD, as well as in MCI, E_2_ levels were reduced compared to healthy controls. Moreover, this finding was accompanied with a correlation of E_2_ and verbal episodic memory delayed recall. So far, our results support formerly published results [18] where in a group of dementia and controls an association between E_2_ and a thai version of the MMSE was reported. However, our results differed from those of Senanarong et al. [18] since they found their results to be valid in men and women while our data could find an association in women only. This might be due to their smaller sample size of AD patients. On the other hand, we were able to, detect group differences in E_2_ level: in Alzheimer's disease and MCI, estrogen level was significantly reduced compared with controls, a finding that confirms a lower E2 level in AD compared to healthy controls [26]. In another cross-sectional study, a higher E_2_ level was associated with a smaller risk to have cognitive impairment [27].

For postmenopausal estrogen users, a lower estimated risk to develop Alzheimer's disease could be found in previous epidemiologic studies [13, 14, 28–32]. However, in a large and well-controlled study, the WHIMS, the combination of estrogen and progesteron failed to prove a beneficial effect, indeed mortality even rose under hormone substitution [16]. More recently, it was shown that E_2_ replacement may be beneficial in ApoE *ε*4 carriers although it did not significantly prevent from dementia for the whole sample [33].

Limitation of our data is the confounding effect of the different age distribution of the three groups although there were no age differences between controls and MCI as our major point of interest. Furthermore, particularly the association of episodic memory and estrogen level was stable to age effects. Episodic memory is regarded as the very early neuropsychological impairment in AD and is considered as the decisive hall-mark in MCI. In previous studies, verbal memory seemed to be the most sensitive cognitive domain to estrogen replacement therapy [17, 34], respectively, the most preserved domain after hysterectomy and eventual E_2_ treatment compared to placebo [35]. In a large review, it was stated from randomized controlled trials that ERT preferentially prevents verbal memory decline in postmenopausal women [36]. Treatment effects in older healthy women are possibly only present in subjects with preserved delayed verbal recall [31]. In summary our findings of a decreased estrogen level in the course of AD as early as in the stage of MCI as well as the findings in the literature of a preponderant positive effect of ERT on verbal memory provide evidence that a reduction of the estrogen level contributes to AD pathogenesis. From neurophysiological studies, several possible biological effects have been described, among others a diminished amyloid-beta toxicity [11, 12], promotion of cholinergic activity in the brain [37], stimulation of axonal spouting and dendritic spine formation [38, 39], and slowing of cerebral atherosclerosis [40].

The advantage of this study was a comparison of E_2_ levels in healthy controls and a group with AD under consideration of MCI patients that are thought to be a group at risk to develop AD. Therefor, early changes that might be relevant for or precede AD might be detectable in MCI. Although there is quite a body of evidence that estrogen is reduced in AD, so far there is a lack of studies in MCI. This is the first study to provide data on E_2_ levels in MCI. Results of our study suggest that E_2_ level is indeed decreased in MCI compared to controls. This reduction may reflect a higher vulnerability to develop AD. Very recently, in men with MCI, a tentative positive effect of hormone replacement therapy could be observed [41].

Finally, a limitation of our study has to be mentioned that it is cross-sectional in design that weakens the conclusions to be drawn from the results.

## Figures and Tables

**Table 1:** Demographic and clinical data of study subjects.

	Controls (*n* = 37)	MCI (*n* = 32)	AD (*n* = 117)	*P* value
Age (years)	60.7 ± 11.3^‡^	65.5 ± 6.9	72.0 ± 8.6^‡^	<0.001
MMSE*	29.2 ± 1.2^‡^	28.1 ± 1.7	21.9 ± 8.6^‡^	<0.001
Gender female/male	17/20	9/23^§^	77/40^§^	<0.001
E_2_ (pg/mL)	39.4 ± 31.3^‡,^	29.8 ± 20.5	21.0 ± 14.5^‡^	<0.001
0 APOE *ε*4-isoallels	84.4%^‡^	75.0%^§^	38.7%^‡,§^	<0.001
1	15.6%	18.8%	48.6%	
2	0%	6.3%	12.6%	
CVLT SD	14.0 ± 2.5^‡,¶^	9.2 ± 3.8^§,¶^	5.2 ± 2.8^‡,§^	<0.001
CVLT LD	13.9 ± 2.6^‡,¶^	9.2 ± 3.9^§,¶^	4.5 ± 2.9^‡,§^	<0.001
CVLT Rec	15.8 ± 0.5^‡,¶^	14.2 ± 1.8^§,¶^	12.4 ± 3.2^‡,§^	<0.001

*Mini-Mental Status Exam.

Significant group differences (post hoc with Bonferroni correction): ^‡^AD versus controls, ^§^AD versus MCI, ^¶^MCI versus controls.
